# Aggregated Hendra virus C-protein activates the NLRP3 inflammasome to induce inflammation

**DOI:** 10.1186/s12950-023-00365-8

**Published:** 2023-11-10

**Authors:** Kristian Barry, Christopher Harpur, Maggie Lam, Michelle D. Tate, Ashley Mansell

**Affiliations:** 1 Centre for Innate Immunity and Infectious Diseases, Hudson Institute of Medical Research, Clayton, VIC Australia; 2 Department of Molecular and Translational Sciences, Monash University, Clayton, VIC Australia

**Keywords:** Hendra, Virus, C-protein, NLRP3, Inflammasome, Inflammation

## Abstract

**Background:**

Hendra virus is an emerging virus with a geographically broad host reservoir. In humans, Hendra virus causes excessive inflammatory disease of the lung and nervous system. Our current understanding as to how Hendra virus or what factors induce inflammation is limited and as such, there are currently no therapeutic options available for patients who contract Hendra virus. Recent studies have identified viral aggregating proteins as drivers of inflammation in influenza A virus and SARS-CoV-2 virus. In this study, we sought to identify potential aggregating Hendra virus proteins as proof-of-concept that inflammasome activation may induce inflammation and contribute to disease pathology.

**Results:**

Here, we have identified that a peptide analogue of Hendra virus C protein (termed HeVc) forms aggregates and activates the NLRP3 inflammasome through phagocytic uptake into cells in vitro. Treatment of cells with the specific NLRP3 inhibitor MCC950 ameliorated IL-1β secretion responses in vitro. Critically, in vivo intranasal inoculation of mice with aggregated HeVc peptide induced pulmonary inflammation, suggesting HeVc may drive immunopathology during infection. Importantly, mice treated with MCC950 demonstrated reduced IL-1β secretion into the bronchoalveolar space, highlighting the role of NLRP3 in host HeV infections and a potential therapeutic strategy to reduce disease pathology.

**Conclusion:**

Taken together, these results identify Hendra virus C protein as a possible contributor to immunopathology during Hendra virus infections. Importantly, these studies highlight a potential role for NLRP3 in driving disease-associated inflammation, critically identifying a possible therapeutic strategy to alleviate disease-associated inflammation of infected patients through targeting of the NLRP3 inflammasome.

**Supplementary Information:**

The online version contains supplementary material available at 10.1186/s12950-023-00365-8.

## Introduction

Hendra virus is an emerging species within the henipavirus genus of the paramyxoviridae family, discovered in 1994 when a spill-over event crossed from its reservoir host of Pteropus bats into horses and humans [1]. Since its discovery, Hendra virus has been reported to have infected 94 horses and 7 humans, with a 60–80% mortality rate in the latter [1]. However, new variants previously undetected in horses have been recently discovered, alluding to more infections in horses that may not have been counted [2]. Due to the large range of infectible hosts and high mortality associated with Hendra virus, and the closely related Nipah Virus, henipaviruses are placed on the WHO list of priority infectious diseases, alongside Severe-acute respiratory Coronavirus (SARS-CoV)-2, Ebola and Zika virus [3]. Although outbreaks of Hendra virus are rare, the increasing urbanization of Australia continues to bring humans and Pteropus bats - Hendra virus’ reservoir host, into proximity [4]. In all human cases of Hendra virus infection, inflammatory cell infiltrates are significant drivers of pathology [5]. Indeed, patients who develop pulmonary symptoms have severe vasculitis with inflammation present in the lungs, kidneys, heart, and the brain with significant inflammatory cell infiltrates into the lung [5]. In animal models, a disease phenotype similar to humans is identified with inflammation occurring in the lungs and central nervous system. Importantly, studies in hamsters have demonstrated that Hendra virus infection induces inflammatory cytokines such as IL-1β, TNF, IL-6, and IFNγ, with IL-1β and TNF speculated to derive from resident microglia cells [6], being shown to play an important role in neural inflammation and disease outcome. Critically however, there is little to no understanding as to how Hendra virus induces this inflammatory phenotype, nor the host immune response.

The NLRP3 inflammasome is an oligomeric protein structure that forms in the cytoplasm in response to a variety of stimuli, including potassium efflux from the cell [7], mitochondrial dysfunction [8] and frustrated phagocytosis of crystalline or aggregate material [9, 10]. The NLRP3 inflammasome consists of the NOD-like receptor NLRP3, an adaptor protein known as Apoptosis associated speck-like protein containing a CARD domain (ASC) and the catalytic enzyme caspase-1. Once formed, the NLRP3 inflammasome is responsible for cleaving the inactive form of the prototypical inflammatory cytokine IL-1β, as well as IL-18, into their bioactive forms for release and function. Due to the range of possible stimuli, NLRP3 has been implicated in a wide range of inflammatory diseases and disorders, including several viral infections such as influenza A virus (IAV). We have previously demonstrated that an aggregated PB1-F2 peptide derived from pathogenic IAV strains induces NLRP3 activation and contributes to disease pathology during infection [11, 12], suggesting that viral protein aggregates may contribute to the immunopathology and inflammation following infection.

Viruses within the paramyxoviridae family all contain a P gene that can be edited through the addition of one or two guanines to generate frameshifts in the resulting mRNA, generating proteins such as the conserved V and W proteins [13]. Furthermore, in some species such as henipaviruses or morbilliviruses, alternate reading of the resulting mRNA can produce a smaller C protein of 15–27 kDa, who’s function varies among paramyxoviruses, with previous studies describing an association with immune evasion [14].

We identified a region of high aggregation potential in the C-terminal of the Hendra virus C protein, subsequently generating a C-protein derived peptide (termed HeVc), that maintains the aggregative potential of the parent protein. In this study, we demonstrate that the HeVc peptide induces activation of the NLRP3 inflammasome in both murine and human macrophages, leading to maturation and secretion of IL-1β. Secretion of IL-1β was ablated in macrophages deficient in components of the NLRP3 inflammasome complex, while treatment of macrophages with the specific NLRP3 inhibitor MCC950 reduced HeVc-induced IL-1β secretion. Critically, mice challenged intranasally with HeVc peptide demonstrated pulmonary inflammation which was reduced in MCC950-treated mice. Together, this study for the first time identifies C protein as the first Hendra virus factor that can activate the host cellular response to infection and highlight the potential therapeutic potential of targeting NLRP3 activity to reduce the inflammatory burden associated with Hendra virus infection.

## Materials and methods

### Cell cultures and reagents

Immortalized murine bone marrow-derived macrophages (iBMDMs; Wildtype, ASC^−/−^, NLRP3^−/−^, Caspase 1^−/−^ and ASC cerulean) were cultured in Dulbecco’s Modified Eagle Medium (DMEM) containing 10% Foetal calf serum (FCS) and 2mM of L-Glutamine at 37^o^C, 5% CO_2_. Human THP-1 cells were cultured in Roswell Park Memorial Institute (RPMI) media containing 10% FCS and 2mM of L-Glutamine. Cells were maintained at 37^o^C/5% CO_2_ for the duration and passaged every 2–3 days.

Human peripheral blood mononuclear cells (hPBMC) were isolated from 30ml of blood using Leucosep tubes (Greiner, 227,290) via the manufacturer’s instructions. In short, mononuclear cells were concentrated using a density gradient solution (Stemcell Technologies, 07801) before treatment with RBC lysis buffer (Sigma-Aldrich, R7757) and resuspension into DMEM containing 10% FCS and 2mM L-Glutamine before immediate use.

LPS-B5 E. coli 055: B5 (Ultrapure; Invivogen) was diluted in Phosphate Buffer Saline (PBS). VX-765, Ac-YVAD-cmk and MCC950 were supplied by Sigma-Aldrich. Latrunculin A was purchased from Caymen Chemicals. HeVc peptide (Amino acid sequence- HeVc: DLLQALREGGVITCQEHTMGMYVLYLIQR; Scrambled: VEVQLGIRGLLIRGMHCTLQDETLYQAMY) was synthesized by Genscript and re-suspended in PBS (2 mg/ml) immediately prior to use.

### Stimulation of macrophages

iBMDMs were seeded into 96-wells (4 × 10^4^ cells per well) and incubated overnight prior to experiments. Human THP-1 cells were seeded into a 96 well plate (6 × 10^4^ cells per well) and treated with 20 ng/mL Phorbol-myristate-13-acetate (PMA) for 12 h before media was replaced and cells were allowed to rest for a further 48 h. hPBMCs were harvested as described above and seeded into a 96-well plate (5 x 10^5^ cells per well) for experiments. All cells were primed with 100ng/ml (iBMDM and THP-1) or 50pg/ml (hPBMC) LPS for 3 h. Cells were then stimulated with either the NLRP3 activators Nigericin (Invivogen, 6 µM) or silica (125 µg/mL), or the AIM2 agonist Poly (dA:dT; Invivogen, 200 ng/ml), or HeVc peptide or scramble control (25–200 µg/ml) for 6 h to promote IL-1β secretion before supernatants were harvested for analysis.

### IL-1β and TNF detection

Detection and assessment of murine and human IL-1β and murine TNF secreted in cell supernatants was conducted by ELISA following manufacturer’s instructions (R&D systems, BD Biosciences). Samples were stored at -80^o^C until analysis.

### IL-1β and Caspase-1 immunoblotting

THP-1 macrophages were seeded in a 12-well plate (5 × 10^5^ cells per well) and treated with 20 ng/ml PMA for 16 h before media was replaced and cells were allowed to rest for a further 48 h. Cells were primed with LPS-B5 (100ng/ml) for 3 h prior to challenge with HeVc peptide (200 µg/ml) or silica (125 µg/ml) in serum free media for 6 h. MCC950 was added 1 h prior to challenge with HeVc and silica respectively. Supernatants were harvested and protein concentrated using Strataclean resin (Agilent). Protein concentrates were then separated on a 4–12% SDS Page gel, and subsequently transferred to a PVDF membrane. IL-1β and Caspase-1 were detected using anti-mouse IL-1β detection biotinylated IgG antibody (R&D Systems) and anti-mouse Caspase-1 monoclonal antibody (Adipogen Life Sciences) where indicated. Proteins were visualized via immunofluorescence using an anti-streptavidin Alexa Fluor 680 conjugate (Life Technologies) and an anti-mouse Alexa Fluor 680 conjugated antibody (Invitrogen).

### ASC cerulean imaging

NLRP3-deficient immortalized macrophages stably expressing ASC-cerulean and NLRP3 were seeded into Ibidi 8-chamber glass bottom slides at 6 × 10^4^ cells per chamber. Cells were then stimulated (or not) with 200 µg/mL of HeVc protein for 5 h, before being fixed with 10% formalin for 15 min, washed with cold PBS and imaged on an Olympus FV1200 confocal microscope. Inflammasome activation was identified as intense ASC-containing specks as imaged as deconvolved z-stacks across 5 randomly selected fields (40x magnification) with the support of Monash Micro Imaging at Monash Health Translation Precinct. ASC specks were denoted as an intense punctate grain within the cell boundaries denoted by brightfield imaging, as well as a lack of diffuse ASC staining within the same cell. ASC-speck containing cells were counted and specks per field were normalised based on the number of cells present in the field.

### Murine intranasal challenge with HeVc peptide

6- to 8-week-old C57BL/6J mice were kept in the Monash Medical Centre animal facility under PC2 containment. All procedures were conducted in accordance with acquired ethics approval from the Monash Medical Centre Animal Ethics Committee Mice were anaesthetised with isoflurane and challenged intranasally with HeVc peptide (50 µg) alone or with MCC950 (5 mg/kg) in a total of 50 µL PBS. Mice were euthanized 6 h post-challenge and bronchial alveolar lavage fluid (BALF) was extracted from the lungs with PBS (3 × 1 ml). BALF was centrifuged (1500 rpm 5 min) to pellet cells which were then treated with RBC lysis buffer (Sigma-Aldrich). The reaction was quenched by washing the cells in FACS buffer (PBS containing 2% (v/v) FBS and 2 mM EDTA). BAL cells were then incubated with fluorescently labelled antibodies in the presence of Fc receptor blocking monoclonal antibody against CD16/CD32 (clone 93, Thermo Fisher Scientific) to limit non-specific antibody binding. BAL cells were stained with monoclonal antibodies to Ly6G (clone 1A8, BD Biosciences) and Ly6C (clone AL-21, BD Biosciences) and the Zombie Aqua viability dye (BioLegend) for 40 min before fixation with 2% PFA for 30 min. Total live BAL cells (Zombie Aqua viability dye^−^) and neutrophils (Ly6G^+^ Ly6C^−^ Zombie Aqua^−^) were quantified using an Aurora Spectral Flow Cytometer (Cytek) and FlowJo™ 10 analysis software (BD Biosciences). Cells were enumerated using a standard amount of blank calibration particles (ProSciTech) as determined using a haemocytometer.

### Statistical analysis

All statistical tests were carried out using Graphpad Prism (v9.1.0). For all tests across multiple groups an Ordinary One-Way ANOVA with Dunnett’s test for multiple comparisons was used. Significance was denoted as a *p*-value below 0.05 (*), 0.01 (**) or 0.001 (***).

## Results

**Fig. 1:**
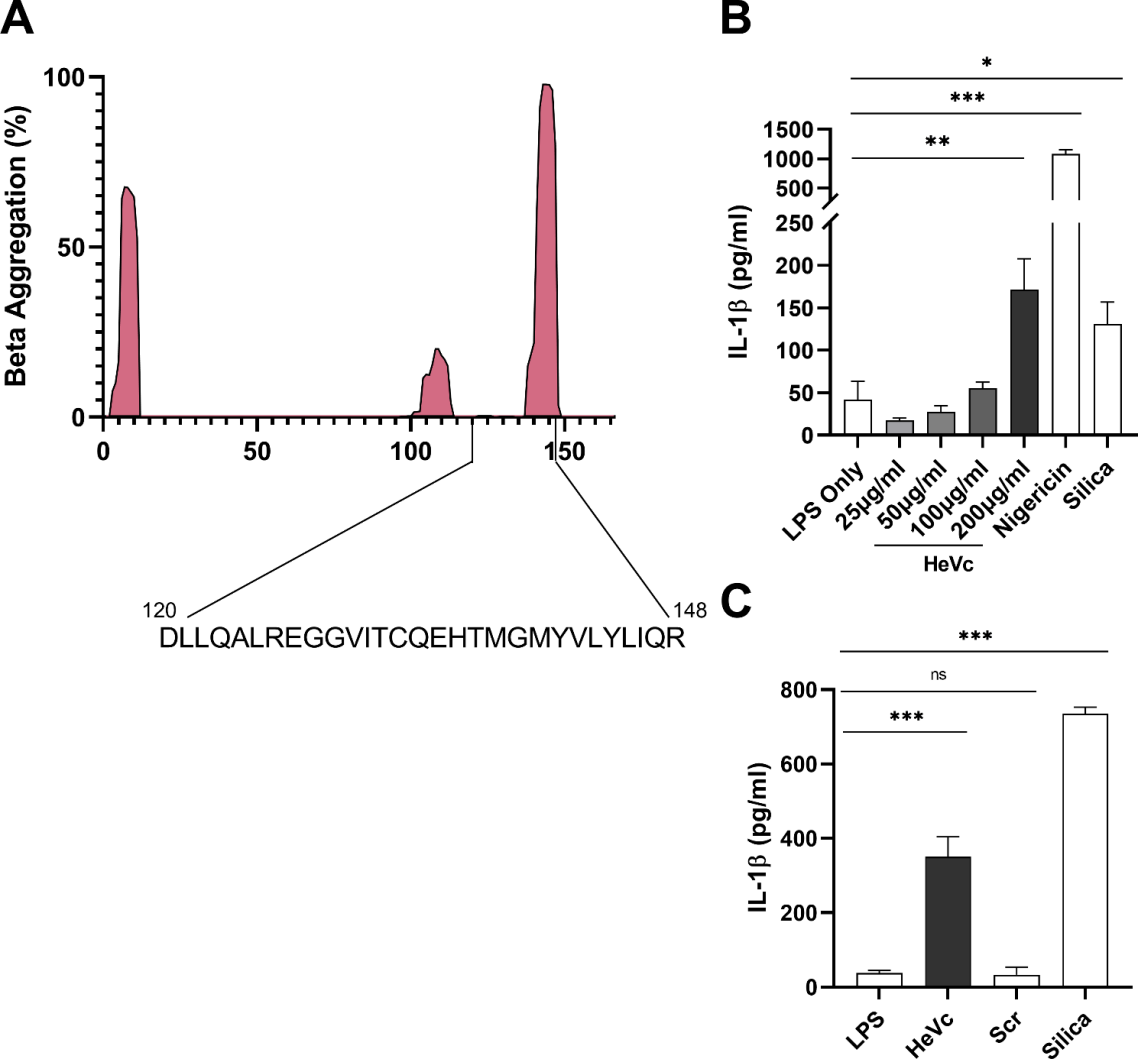
HeVc peptide induces IL-1β secretion in murine immortalized macrophages. **(A)** Beta aggregation score of C-protein amino acids as derived by Tango software alongside a representative image of where the HeVc peptide is taken from the main C-protein. **(B)** Murine iBMDMs were seeded at 4 × 10^4^ per well 20 h prior to priming with 100 ng/ml LPS-B5 for 3 h and then challenged with aggregated HeVc peptide (25–200 µg/ml), or NLRP3 stimulants (6 µM Nigericin or 125 µg/ml Silica) as indicated for an additional 6 h. Supernatants were harvested and assayed for IL-1β by ELISA according to manufacturer’s instructions. Results shown are representative of 3 independent experiments conducted in triplicate, presented as mean ± SD where appropriate, *p < 0.05, **p < 0.01, ***p < 0.001. Ordinary One-way ANOVA with Dunnett’s test for multiple comparisons

We have previously shown that the IAV aggregating protein PB1-F2 can elicit an NLRP3 dependent inflammasome response contributing to IAV disease pathogenesis [11, 12]. Therefore, we hypothesized that other viral aggregating proteins may drive inflammasome activity and contribute to disease inflammation. Through *in-silico* analysis of other viral proteins, we identified that the C-protein of Hendra virus (HeVc), had high aggregative potential similar to that of IAV PB1-F2. Furthermore, the C-proteins of paramyxoviruses, are expressed in an alternate reading frame and have immunomodulatory effects when in the cytosol of cells, similarly to that previously described for PB1-F2 [14]. Therefore, we sought to identify whether extracellular protein aggregates of Hendra virus C-protein would be effective at inflammasome activation. As Hendra virus is a biosafety level 4 pathogen we derived a peptide from HeVc protein containing the N-terminus portion aggregating potential for further experiments (Fig. 1A), which we visually confirmed formed aggregates in PBS (Supplemental Fig. 1).

### HeVc peptide induces IL-1β secretion in murine macrophages through NLRP3 inflammasome activation

To determine whether the HeVc peptide was able to induce IL-1β secretion and possible inflammasome activation, we challenged immortalized murine bone marrow-derived macrophages (iBMDMs) with a range of concentrations of HeVc peptide, or with the well-described NLRP3 inflammasome activators silica and nigericin for 6 h as controls. As can be seen in Fig. 1B, HeVc peptide significantly induced IL-1β secretion in iBMDMs at 200 µg/mL, as was also observed for nigericin and silica respectively. Furthermore, treatment with a scrambled soluble HeVc peptide (Fig. 1C) did not induce significant IL-1β secretion, highlighting the insoluble aggregating nature of HeVc peptide is required for IL-1β secretion.

**Fig. 2:**
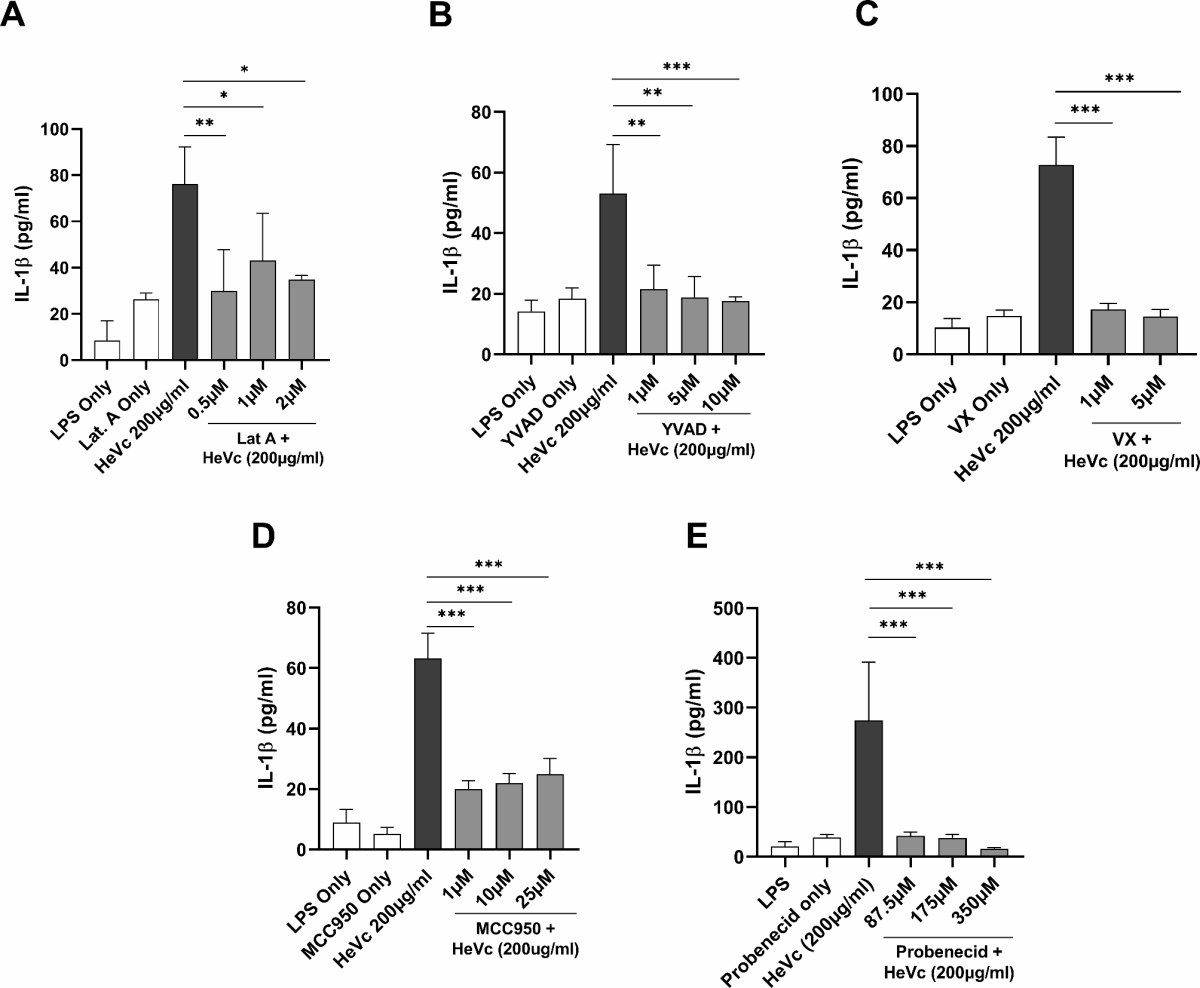
HeVc peptide-induced IL-1β secretion requires phagocytosis, caspase-1 activation and NLRP3 function. Murine iBMDMs were primed with 100 ng/ml LPS-B5 for 2 h before inhibitors of **A**) phagocytosis (0.5-2 µM Latrunculin A), **B-C**) caspase-1 activity (1–10 µM Ac-YVAD-cmk, 1–5 µM VX695) and **D-E**) NLRP3 (1–25 µM MCC950, 87.5-250 µM Probenecid) and were added to cell media for 1 h. Cells were then stimulated with 200 µg/ml of HeVc peptide for 6 h and supernatants were harvested for the detection of IL-1β secretion via ELISA. Results are representative of 3 independent experiments conducted in triplicate and are presented as mean ± SD. *p < 0.05, **p < 0.01, ***p < 0.001, Ordinary One-way ANOVA with Dunnett’s test for multiple comparisons

The phagocytosis of disease- and infection-related protein or peptide aggregates by macrophages, is a well characterized inducer of NLRP3 inflammasome activation [9–11]. As can be seen in Fig. 2A, inhibition of phagocytosis with Latrunculin A inhibited HeVc-induced IL-1β secretion, while targeting of caspase-1 catalytic activity with Ac-YVAD-cmk (Fig. 2B) or VX765 (Fig. 2C), similarly significantly reduced IL-1β secretion. Finally, MCC950, a specific small molecule NLRP3 inhibitor inhibited IL-1β secretion (Fig. 2D) commensurate with probenecid (Fig. 2E), which has previously been demonstrated to target aggregated IAV PB1-F2 NLRP3 inflammasome activation [12, 15].

Taken together, these results demonstrate HeVc peptide requires phagocytosis to induce activation of an NLRP3 inflammasome to mediate caspase-1-mediated enzymatic IL-1β secretion in both mouse and human macrophages.

**Fig. 3:**
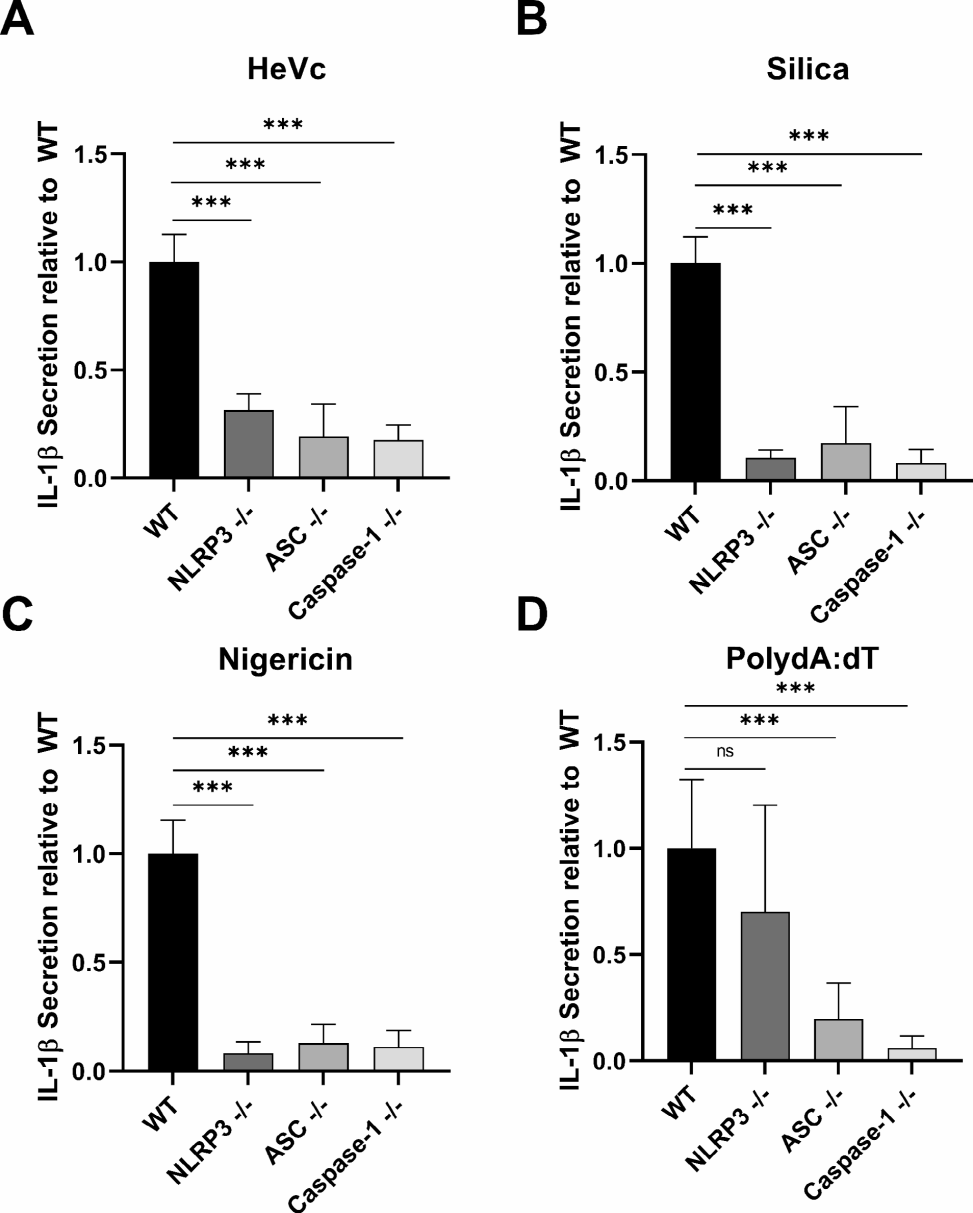
HeVc peptide-induced IL-1β secretion requires an NLRP3, ASC and Caspase-1 inflammasome complex. Murine iBMDMs deficient in either NLRP3, ASC, or Caspase-1 were primed with 100 ng/ml LPS-B5 for 3 h before being stimulated with **A**) HeVc peptide (200 µg/ml), **B**) Silica (125 µg/ml), **C**) Nigericin (6 µM) or **D**) poly (dA:dT) for 6 h and supernatants were harvested and assayed for IL-1β secretion by ELISA. Results are pooled and normalized to wildtype response from 3 independent experiments conducted in triplicate and are presented as mean ± SD. *p < 0.05, **p < 0.01, ***p < 0.001, Ordinary One-way ANOVA with Dunnett’s test for multiple comparisons

To further determine whether the NLRP3 inflammasome was the major driver of the response to extracellular HeVc peptide, we examined IL-1β secretion from iBMDM cells individually deficient in NLRP3, the adaptor protein ASC or Caspase-1 following aggregated HeVc peptide challenge. Clearly highlighting the requirement of NLRP3, ASC, or caspase-1 for HeVc peptide to activate an inflammasome complex, IL-1β secretion was significantly attenuated in the absence of all NLRP3 inflammasome components (Fig. 3A), compared to wildtype (WT) macrophages. Critically, responses to known NLRP3 agonists Nigericin (Fig. 3C) and silica (Fig. 3B) were also attenuated, while induction of the AIM2 inflammasome with poly (dA:dT), was able to induce IL-1β secretion in NLRP3-deficient cell lines, but not ASC or Caspase-1 deficient iBMDMs (Fig. 3D), suggesting a specificity for NLRP3 activation for HeVc peptide. Together, these results identify that aggregated HeVc peptide induces the activation of an NLRP3 inflammasome complex that leads to IL-1β secretion from macrophages.

### HeVc peptide induces ASC-speck formation in ASC-cerulean cell lines

**Fig. 4:**
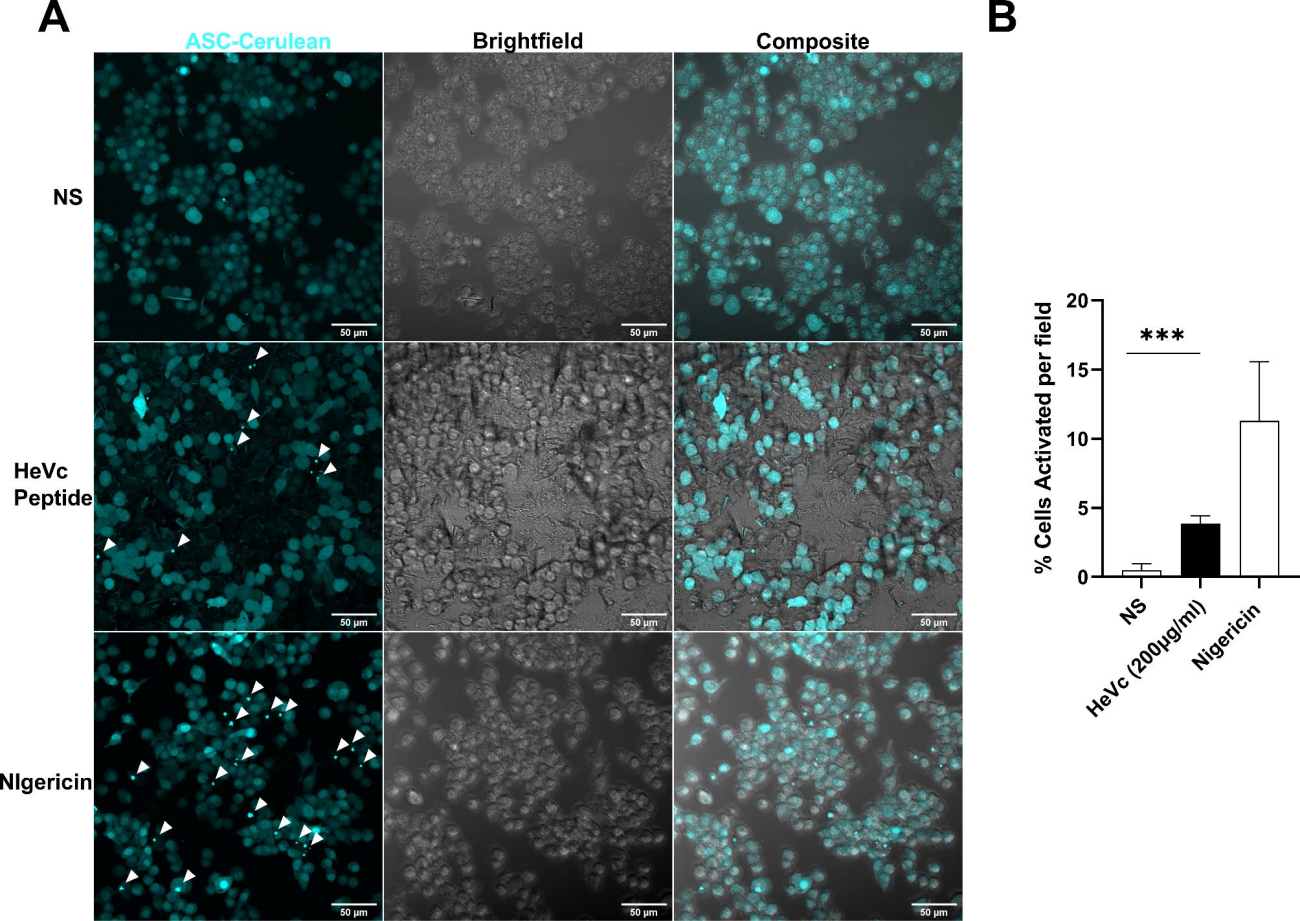
HeVc peptide induces ASC speck formation in ASC-cerulean iBMDMs. Murine ASC-cerulean iBMDMs were seeded in ibidi 8 chamber slides at 6 × 10^4^ cells per well 24 h prior to stimulation with 200 µg/ml HeVc peptide for 5 h or Nigericin (6 µM) for 1 h before being fixed with 10% formalin solution and imaged on an FV1200 confocal microscope at 40x objective. Five random z-stacked fields per treatment were taken per condition and the number of cells identified in brightfield and number of ASC specks were quantified using FIJI. Images are representative of 3 independent experiments. Results in **(B)** are triplicate and are representative of 3 independent experiments and are presented as mean ± SD. ***p < 0.001 Ordinary One-way ANOVA with Dunnett’s test for multiple comparisons

As we had determined that HeVc peptide required NLRP3 inflammasome activation for eliciting IL-1β secretion in iBMDMs, we next sought to visualize inflammasome activation through ASC-speck formation. NLRP3-deficient iBMDMs reconstituted with ASC-cerulean and NLRP3-Flag were stimulated with HeVc peptide or Nigericin and ASC-speck formation observed using confocal microscopy. As can be observed in Fig. 4A, while no to limited specks were observed in unchallenged iBMDMs, HeVc peptide was able to induce ASC speck formation (Fig. 4A, ASC-cerulean, white arrows) after 5 h of challenge in the cytosol of cells, indicative of formation of an inflammasome complex, similar to that observed for Nigericin (bottom panels). Quantification of cytosolic ASC specks (Fig. 4B) demonstrated that both HeVc peptide and Nigericin, induced significantly increased inflammasome complex formation compared to untreated cells. These observations further highlight NLRP3 inflammasome formation in response to HeVc peptide and expands the role of the NLRP3 inflammasome in IL-1β secretion in response to extracellular HeVc peptide.

### HeVc peptide induces IL-1β secretion in human THP-1 derived macrophages and human peripheral blood mononuclear cells that is reduced by NLRP3 inhibition

**Fig. 5:**
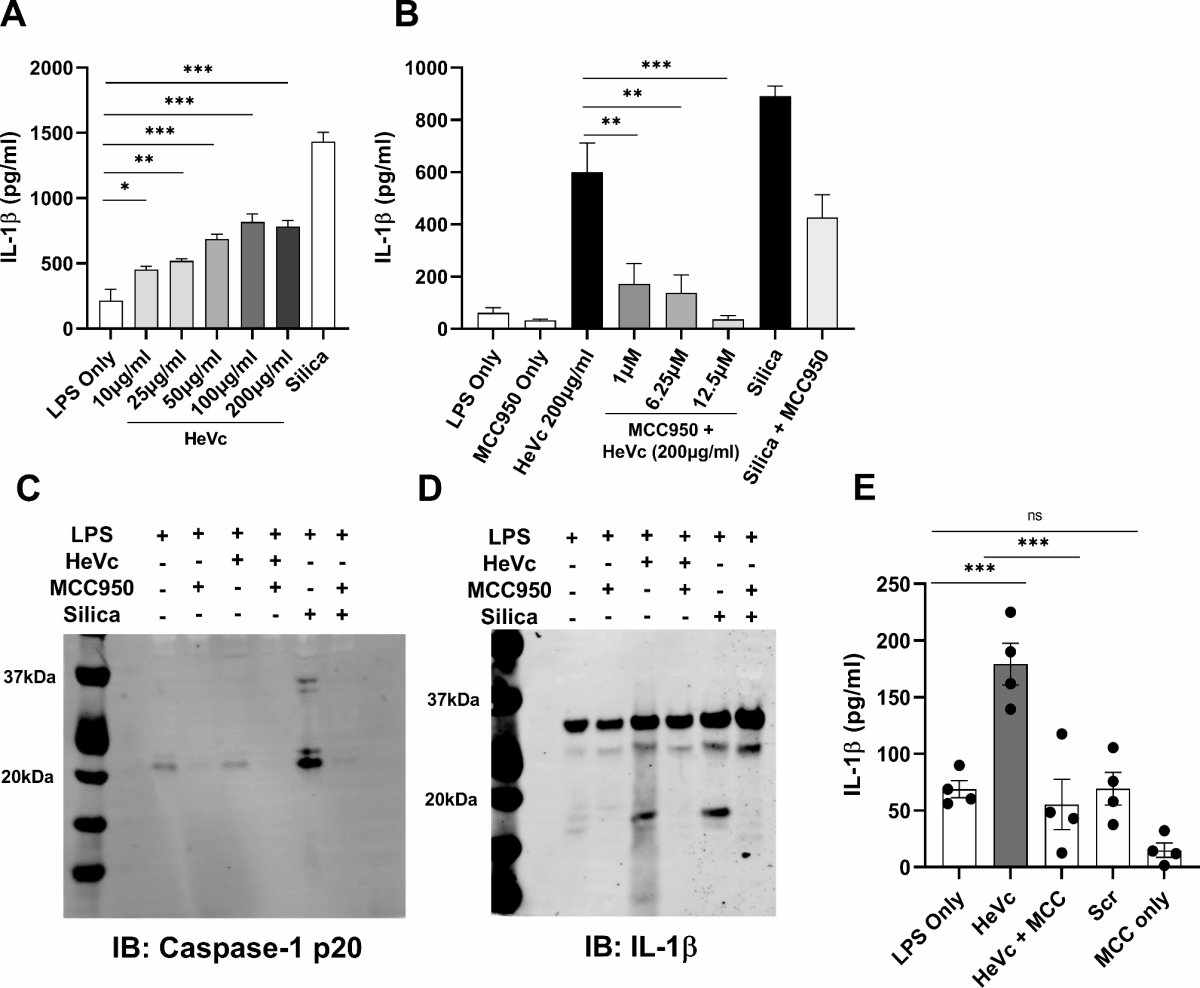
HeVc peptide-induces maturation of IL-1β and caspase-1 in human THP-1 macrophages and PBMCs. Human THP-1 cells seeded in a 12-well plate (5 × 10^5^ cells per well) were differentiated with 4 µM PMA in media for 16 h before their media was changed and incubated for a further 48 h to differentiate into macrophages. THP-1 derived macrophages were primed with 100 ng/ml LPS-B5 for 3 h in the presence or not of **B)** MCC950 and **A-B)** stimulated with 200 µg/ml of HeVc peptide for 6 h. Supernatants were harvested for the detection of IL-1β secretion via ELISA. **C-D)** THP-1 macrophages were treated with MCC950 or not where indicated and challenged with aggregated HeVc peptide or silica for 6 h respectively. Supernatants were harvested and proteins separated on a 4–12% SDS-PAGE gel before visualization of IL-1β and caspase-1 was conducted by immunoblot. **E)** hPBMCs from 4 donors were separated from 30 ml blood via density-gradient separation and seeded into a 96-well plate (2.5 × 10^5^ cells per well). hPBMCs were primed for 3 h with 50 pg/ml LPS in the presence or not of MCC950 before stimulation with HeVc peptide (200 µg/ml) or Scramble (200 µg/ml) for 6 h. Supernatant was analysed via ELISA for IL-1β secretion. **A-B)** Results are representative of 3 independent experiments conducted in triplicate and are presented as mean ± SD. **E)** Results are the mean ± SEM of four independent donors. Ordinary One-way ANOVA with Dunnett’s test for multiple comparisons *p < 0.05, **p < 0.01, ***p < 0.001

Given the inflammatory response in humans contributes to disease pathology and mortality, we next sought to determine whether HeVc peptide was able to activate the NLRP3 inflammasome in human macrophages and generate mature IL-1β. Human THP-1 differentiated macrophages were primed with LPS prior to stimulation with a range of aggregated HeVc peptide concentrations. As can be seen in Fig. 5A, HeVc peptide elicited a dose-dependent secretion of IL-1β, which was dose-dependently inhibited by MCC950 treatment (Fig. 5B); similar to our results in murine macrophages (Fig. 2). To confirm that HeVc-induced maturation of caspase-1 and IL-1β, we next verified by immunoblot that HeVc-induced both caspase-1 (Fig. 5C) and IL-1β (Fig. 5D) maturation into their bioactive p20 and p17 forms respectively, which critically, was inhibited with MCC950. These observations would reinforce that aggregated HeVc peptide induces an NLRP3 inflammasome leading to release of bioactive IL-1β in human macrophages. To validate that HeVc peptide may also enhance inflammatory events in human cells, we examined cellular responses in LPS-primed human peripheral blood mononuclear cells (hPBMCs) exposed to HeVc peptide as compared to scrambled peptide. As can be seen in Fig. 5E, while HeVc peptide induced IL-1β secretion from hPBMCs, scrambled peptide did not. Importantly, treatment with MCC950 significantly inhibited this secretion, suggesting HeVc peptide activated the NLRP3 inflammasome.

### HeVc peptide induces pulmonary inflammation in an in vivo challenge model

**Fig. 6:**
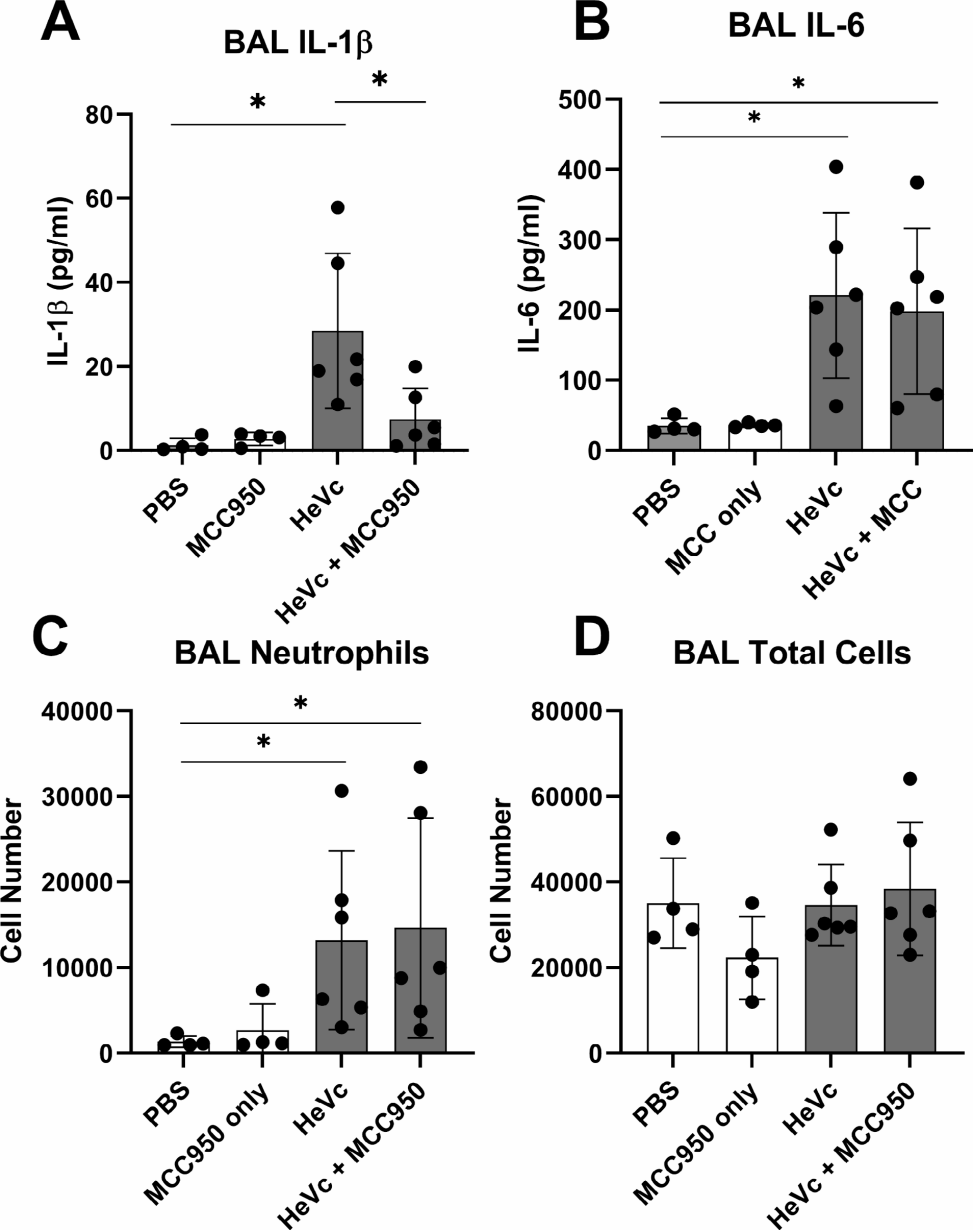
HeVc peptide induces inflammation and NLRP3-dependent IL-1β secretion in the lungs of mice. Wildtype C57BL/6 mice were intranasally challenged with 50 µg HeVc peptide in PBS, 50 µg of HeVc peptide and 100 µg (5 mg/kg) MCC950, or PBS alone for 6 h. Bronchoalveolar lavage was harvested. **A**) IL-1β and **B**) IL-6 secretion was analysed by ELISA of lavage supernatants. Total **C**) neutrophil cell numbers as determined by Ly6G^+^ Ly6C^−^ cells, and C) BAL cell counts, determined by flow cytometry. Results are representative of 6 mice per group shown as individual circles. Data presented as mean ± SD. *p < 0.05. Ordinary One-way ANOVA with Dunnett’s test for multiple comparisons

To identify whether HeVc peptide was able to activate the NLRP3 inflammasome in an in vivo setting, we intranasally challenged C57BL/6 mice with HeVc peptide and assessed cellular infiltrates and the secretion of IL-1β through bronchoalveolar lavage (BAL) analysis. Mice challenged with aggregated HeVc peptide for 6 h showed a significant increase in IL-1β secreted into the bronchoalveolar space (Fig. 6A). Critically, intranasal treatment with the NLRP3 inhibitor MCC950 [16] significantly reduced IL-1β in BAL fluid, suggesting that IL-1β secretion in response to HeVc peptide is driven by the NLRP3 inflammasome. Conversely, while IL-6 was increased in response to aggregated peptide challenge, MCC950 did not reduce IL-6 secretion into the BALF, further highlighting the specific nature of the immune response to aggregated peptide via NLRP3. Inflammation in the lung was further highlighted by a significant increase of neutrophils in the bronchoalveolar space (Fig. 6C). Importantly, treatment with MCC950 did not reduce the number of neutrophils and IL-6 (Fig. 6B-C), highlighting its specificity in inhibiting the NLRP3 inflammasome and not due to reduced cellular infiltrates due to decreased IL-1β secretion. Consistent with this, total lung cell counts (Fig. 6D) were unchanged between all groups. Taken together, these data highlight the propensity for HeVc peptide, and thus Hendra virus C-protein, to initiate an inflammatory response *in-vivo* through the activation of the NLRP3 inflammasome. Critically, we also demonstrate that IL-1β secretion into the pulmonary space can be inhibited with NLRP3 inhibitors, highlighting the possibility of targeting NLRP3 activity to reduce pulmonary inflammation during Hendra Virus infection.

## Discussion

Henipaviruses pose a unique threat to human health, with a range of possible hosts and high mortality associated with human infection [1]. Hendra virus infection in animal models has been demonstrated to induce detrimental inflammatory infiltrates associated with IL-1β and TNF in the lung and nervous system [17], although the viral drivers of inflammation in infected individuals is poorly understood. Recent studies have demonstrated a critical role of inflammasome complexes in driving the pathology during inflammatory viral diseases such as IAV, SARS CoV2, respiratory syncytial virus and *Flaviviridae* [18, 19]. Our study serves as a proof of concept model identifying aggregated C protein as a possible novel agonist for NLRP3 inflammasome activation and IL-1β secretion during Hendra virus infection that may contribute to increased detrimental inflammation during in vivo infection.

Aggregates derived from viral proteins are well known and are often associated with assisting in replication or avoiding host defences [20]. Previous studies have identified viral aggregates as activators of the NLRP3 inflammasome in Influenza A virus [11, 12] through uptake of extracellular aggregates, and recently, ORF8b protein from SARS-CoV has also been shown to form aggregates when expressed in cells and is able to induce activation of the NLRP3 inflammasome [21]. This study therefore further enhances the concept that aggregated viral proteins may constitute a novel sub-family of NLRP3 agonists that may contribute to increased disease pathology.

While no previous studies investigating Hendra virus C protein have been described to the best of our knowledge, studies into the Nipah virus C protein (of which Hendra virus C protein shares over 80% homology) identify that it is responsible for immune evasion and inhibition of interferon [22] and replication [23]. C-proteins of other paramyxoviruses have also been demonstrated to have immune evasion roles involved in interferon regulation [14].Importantly, this draws a parallel with highly pathogenic IAV-expressed PB1-F2, which is responsible for immune modulation and interferon inhibition [24], while also being able to activate the NLRP3 inflammasome [11, 12]. Furthermore, SARS-CoV ORF8b, which has been shown to activate the NLRP3 inflammasome [21] also had previously been identified to inhibit interferon signaling [25]. Therefore, NLRP3 inflammasome activation may be an evolutionary tactic for the host to detect viral aggregate proteins that are altering the interferon response, as demonstrated in PB1-F2, ORF8b and HeVc.

Interestingly, another virus within the henipavirus genus, known as Cedar virus, lacks the cellular machinery or coding capacity to produce the P-gene alternate reading frames for the V, W and C proteins [26]. Importantly, Cedar virus infection is considered non-pathogenic, with viral replication and neutralizing antibodies produced with no inflammatory disease phenotype or overt cytokine production in ferrets or guinea pigs [26]. Cedar virus therefore highlights the importance of these alternate reading frame proteins in immune-driven pathology during infection. Additionally, further work with the closely related Nipah virus C protein – of which Hendra virus C protein shares 83.2% amino acid homolog, may provide insight into its functions. Indeed, Nipah virus lacking C protein has been demonstrated to have reduced replicative potential *in-vitro* [23] which may be attributed to possible C protein control of genome replication [27]. Indeed, Nipah virus C protein, as well as the P, V and W proteins has been shown to interfere with IFNα/β induction in a plasmid vector system [28]. Nipah virus V protein has also been shown to accomplish this through the sequestration of STAT1/STAT2 [23, 29], although knockout of C protein failed to replicate STAT1 sequestration [23], and therefore may attenuate IFNα/β expression through a different unknown pathway. Supporting this, attenuation of Nipah virus C protein in *in-vivo* infection of hamsters also significantly reduced lethality alongside an increase in infiltrating inflammatory cells and the production of inflammatory cytokines, suggesting a role for immune control for Nipah virus C protein and thus has been extrapolated as a possible function for Hendra virus C proteins [23]. Comparatively, analogous immunomodulatory targeting of STAT proteins has been demonstrated for IAV PB1-F2, further highlighting the similarities between modulation of the interferon response by viral factors that may also act as host ‘alarmins’. Further study into the immunomodulatory roles of Nipah and Hendra C protein may therefore complement our study to further elucidate the roles of these proteins during their respective infections.

In our study we have shown that inhibition of NLRP3 in mice challenged with HeVc peptide alleviated IL-1β secretion in the lung and thus possibly highlighting Hendra Virus C protein as a contributor to inflammation. Therefore, controlling NLRP3 activation may prove a beneficial therapeutic for Hendra virus infection. However, temporal inhibition of NLRP3 with MCC950 has been shown to have detrimental effects on viral infection if induced too early in infection, highlighting the requirement for NLRP3 to induce a protective immune response [30]. Consequently, partial inhibition of NLRP3, or inhibition of NLRP3 in targeted cell types during infection could retain NLRP3’s protective effects while dampening detrimental inflammation. This is supported by previous work in our group whereby the lower affinity inhibitors of NLRP3 activation Probenecid and AZ11645373 were able to alleviate IAV infection severity without requiring temporal control [15]. Therefore, it may be possible that partial NLRP3 inhibition may also serve to reduce the severity of Hendra virus infection by dampening C-protein derived inflammation, similarly to previous studies in IAV.

In conclusion, we have demonstrated a possible novel role of the Hendra virus C protein in activating the NLRP3 inflammasome and contributing to immunopathology in vitro and in vivo. This proof-of-concept study identifies C protein as a potential immunogenic factor for Hendra virus-associated inflammatory disease. Furthermore, we highlight that through inhibition of NLRP3 during HeVc peptide challenge, IL-1β secretion into BAL can be reduced, which may alleviate pulmonary inflammation, providing a potential novel therapeutic strategy where currently none exist. These studies further enhance viral aggregates as immune agonist of the NLRP3 inflammasome, highlighting that viral aggregate proteins may constitute a new family of pathogen-associated molecular patterns that play a significant role in the host response to infection, but alternatively, may contribute to the hyperinflammatory pathophysiology associated with diseases such as avian influenza, SARS CoV and Hendra virus. Future work investigating C-protein driven NLRP3 activation in virally infected cells, as well as Hendra virus infection models with C-deficient virus may further uncover the role of NLRP3 in *in-vivo* immune activation during Hendra virus infection.

### Electronic supplementary material

Below is the link to the electronic supplementary material.

Supplementary Material 1

## Data Availability

The datasets used and/or analysed during the current study are available from the corresponding author on reasonable request.
