# Supplementary material for: Aggregated Hendra virus C-protein activates the NLRP3 inflammasome to induce inflammation
Source: J Inflamm (Lond). 2023 Nov 10;20:38. doi: 10.1186/s12950-023-00365-8 (PMC10636811; doi:10.1186/s12950-023-00365-8)
Supplement: Supplementary file 1 — Supplementary Material 1 [file 12950_2023_365_MOESM1_ESM.docx]

**Supplementary Material**

**Aggregated Hendra virus C-protein activates the NLRP3 inflammasome to induce inflammation**

Kristian Barry^1,2^, Christopher Harpur^1,2^, Maggie Lam^1,2^, Michelle D. Tate^1,2^, Ashley Mansell^1,2^

^1^Centre for Innate Immunity and Infectious Diseases, Hudson Institute of Medical Research, Clayton, VIC, Australia

^2^Department of Molecular and Translational Sciences, Monash University, Clayton, VIC, Australia


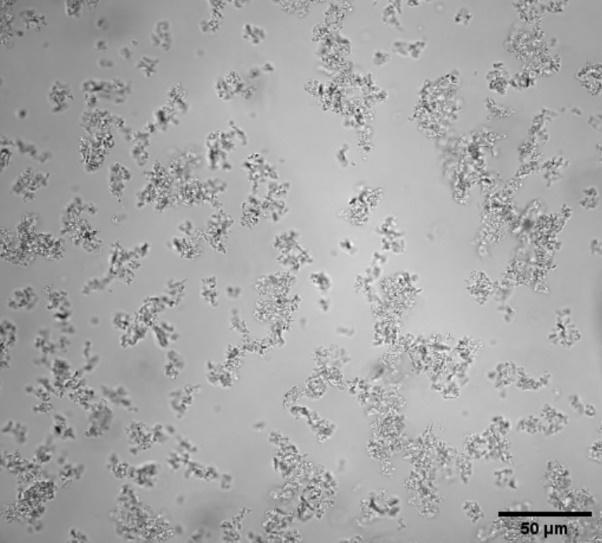

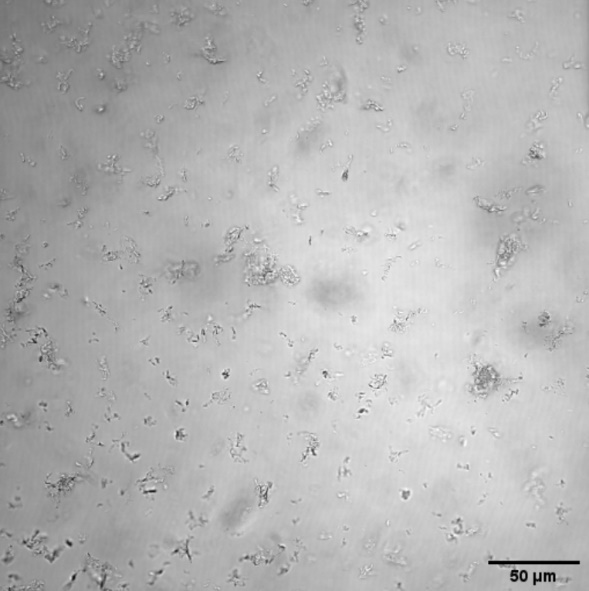


**A**

**B**

**Supplemental Material 1. HeVc and Scramble peptide aggregation images**

**A)** HeVc peptide and **B)** Scramble peptide were reconstituted with PBS at 2 mg/ml and placed into an Ibidi 8-chamber slide for imaging using an FV1200 microscope on brightfield setting at 40x objective. Images representative of 5 random fields within each chamber.


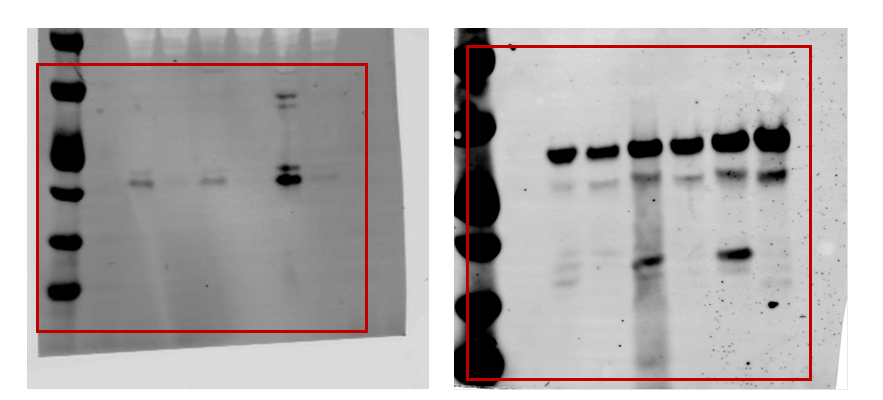


**Supplemental Material 2. Full length blots of HeVc-treated human macrophages.**

THP-1 macrophages (1 x 10^6^) were treated with MCC950 or not where indicated and challenged with aggregated HeVc peptide or silica for 6 hrs respectively. Supernatants were harvested and proteins separated on a 4-12% SDS-PAGE gel before visualization of IL-1β and caspase-1 was conducted by immunoblot. Results are representative of 3 independent experiments. Red box defines image used in Figure 5C and 5D respectively.
